# Supplementary material for: Small-sample learning reveals propionylation in determining global protein homeostasis
Source: Nat Commun. 2023 May 17;14:2813. doi: 10.1038/s41467-023-38414-8 (PMC10192394; doi:10.1038/s41467-023-38414-8)
Supplement: Supplementary file 1 — Supplementary Information [file 41467_2023_38414_MOESM1_ESM.pdf]

Supplementary Information:

Small-sample learning reveals propionylation in determining global protein homeostasis

Ke Shui[1,†], Chenwei Wang[1,†], Xuedi Zhang[2], Shanshan Ma[1], Qinyu Li[1], Wanshan Ning[1], Weizhi Zhang[1], Miaomiao Chen[1], Di Peng[1], Hui Hu[1], Zheng Fang[3], Anyuan Guo[1], Guanjun Gao[2], Mingliang Ye[3], Luoying Zhang[1,4]*, Yu Xue[1,5]*

[1]Key Laboratory of Molecular Biophysics of Ministry of Education, Hubei Bioinformatics and Molecular Imaging Key Laboratory, Center for Artificial Intelligence Biology, College of Life Science and Technology, Huazhong University of Science and Technology, Wuhan, Hubei 430074, China

[2]School of Life Science and Technology, ShanghaiTech University, 393 Middle Huaxia Road, Shanghai 201210, China

[3]CAS Key Laboratory of Separation Science for Analytical Chemistry, Dalian Institute of Chemical Physics, Chinese Academy of Sciences, Dalian, 116023, China

[4]Hubei Province Key Laboratory of Oral and Maxillofacial Development and Regeneration, Wuhan, Hubei 430022, China

[5]Nanjing University Institute of Artificial Intelligence Biomedicine, Nanjing, Jiangsu 210031, China

[†]These authors contributed equally to this work.

*To whom correspondence should be addressed.

Yu Xue, E-mail: xueyu@hust.edu.cn.

Luoying Zhang, E-mail: zhangluoying@hust.edu.cn.

Index

Supplementary Information: 1

Supplementary Discussion 3

Supplementary Figures 6

Supplementary References 27

Supplementary Discussion

Post-translational modifications (PTMs) provide an important means to dynamically and reversibly regulate the proteostasis network (PN)[1, 2, 3, 4]. Besides lysine acetylation (Kac)[1, 5], protein phosphorylation is also involved in regulating proteostasis[1, 2, 3, 6]. For example, phosphorylation of the α subunit of eukaryotic initiation factor 2 (eIF2α) inhibits protein translation and adjusts proteostasis in response to various cellular stresses, such as accumulation of misfolded proteins in the endoplasmic reticulum (ER)[2, 3, 7]. Moreover, phosphorylation of molecular chaperones such as heat shock protein 70 (HSP70) and 90 (HSP90) constitutes the "chaperone code", and dramatically changes the chaperone activity and selectivity upon recognizing the protein substrates[8, 9, 10]. In addition, ubiquitination is also a critical PTM involved in proteostasis, and the first discovered ubiquitination substrate, cyclin, is specifically degraded by the UPS pathway to inactivate cyclin-dependent kinase 1 (CDK1) at the end of mitosis[11, 12]. In this study, we uncovered a functionally important lysine propionylation (Kpr) site, H2BK17pr, which participates in determining global protein level. This provides an additional layer of proteostasis regulation.

Previous studies have demonstrated that PTMs undergo weak functional constraints, and up to 65% of phosphorylation sites (p-sites) are estimated to be non-functional[13, 14]. Beltrao et al. collected ~200,000 phosphorylation, acetylation and ubiquitination sites from 11 eukaryotic organisms, and computationally revealed that functionally important PTM sites are evolutionarily more conserved and preferentially involved in regulating PTM crosstalk events, functional domain activity and PPIs[15]. Taking 2,638 functionally important p-sites as the training data set and using traditional machine learning methods including RF and gradient-boosting machine, Beltrao et al. developed funscoR, a computational program that integrated 59 types of proteomic, structural, regulatory and evolutionary features to predict the functional relevance of p-sites [16]. Under a cut-off value of 0.5, they predicted ~10% of the phosphoproteome to be functionally important. Recently, we also developed a machine learning-based method called inference of functional phosphosites (iFPS), which integrated 6 types of

sequence and structure features and used 121 known worm p-sites with important functions for model training [17]. Using iFPS, we prioritized 25 of > 15,000 worm p-sites to be potentially involved in longevity, and successfully discovered 3 p-sites that indeed regulate lifespan.

Through our propionylomic profiling, we quantified 344 Kpr sites in 171 proteins from fly heads. Based on our current understanding of the physiological roles of PTMs, we hypothesized that only a small subset of the Kpr sites identified are functionally important. However, traditional machine learning strategies mentioned above are not applicable in this study because only 13 Kpr sites have been validated to be functional (Supplementary Data 2c). Training a model directly using such a small data set will be highly instable and error-prone, and undoubtedly lead to over-fitting. To resolve this problem, we adopted the state-of-the-art small-sample learning method of Model-Agnostic Meta-Learning (MAML)[18], coupled with traditional machine learning and deep learning methods. In KprFunc, an initial model was trained to predict *bona fide* Kpr sites from protein sequences (Fig. 2a). Using MAML, this initial model was fine-tuned to predict the functional relevance of Kpr sites based on the 13 Kpr sites with known functions. Such an integrative framework achieved promising accuracy values on both tasks, and helped us to prioritize and validate H2BK17pr as a functionally important and conserved propionylation event. We believe with some modifications this framework can be easily adapted to help resolve other questions in biological research that has not been studied much previously and has little relevant information.

Besides KprFunc, three computational methods were previously developed for prediction of general Kpr sites. In 2017, Ju et al. obtained 413 experimentally identified Kpr sites in 192 proteins [19] from our protein lysine modification database (PLMD 3.0)[20], an earlier version of the Compendium of Protein Lysine Modifications (CPLM 4.0)[21]. After redundant and homologous clearance, they compiled a benchmark data set of 327 Kpr sites in 164 proteins. Four types of sequence features, including amino acid composition (AAC), amino acid factors (AAF) of various physicochemical properties in the AAIndex database, binary encoding (BE), and composition of *k*-spaced amino acid pairs (CKSAAP), were integrated and the computational model was trained by the

biased support vector machine (SVM). Then they developed an online service of PropPred for prediction of Kpr sites, with an area under the curve (AUC) value of 0.7966 from the 10-fold cross-validation[19]. In addition, Wang et al. collected 1,782 known and non-redundant Kpr sites in 820 prokaryotic proteins, and developed PropSeek for specifically predicting prokaryotic Kpr sites[22]. Six types of sequence features were encoded and integrated, while the SVM method was used for model training. The 10-fold cross-validation was performed on various data sets, with AUC values ranging from 0.798 to 0.845. Recently, Li et al. implemented a strategy of deep learning followed by transfer learning[23]. They obtained 9,584 malonylation sites in 3,429 proteins from our PLMD 3.0[20], and then used a deep learning framework of recurrent neural network (RNN) to construct a computational model for predicting malonylation sites. Then, they obtained 408 Kpr sites in 189 proteins from PLMD 3.0 and the literature. The initial RNN model was used for feature extraction from the Kpr sites, and SVM was used to train the final model. Through the 10-fold cross-validation, such a strategy achieved an AUC value of 0.8088, which was comparable to PropPred. The three methods were not developed for the purpose of predicting the functional relevance of Kpr sites. Moreover, the update of CPLM 4.0 renders more Kpr sites available in this study for pre-training a more reliable model to capture the sequence features of Kpr sites.

Supplementary Figures

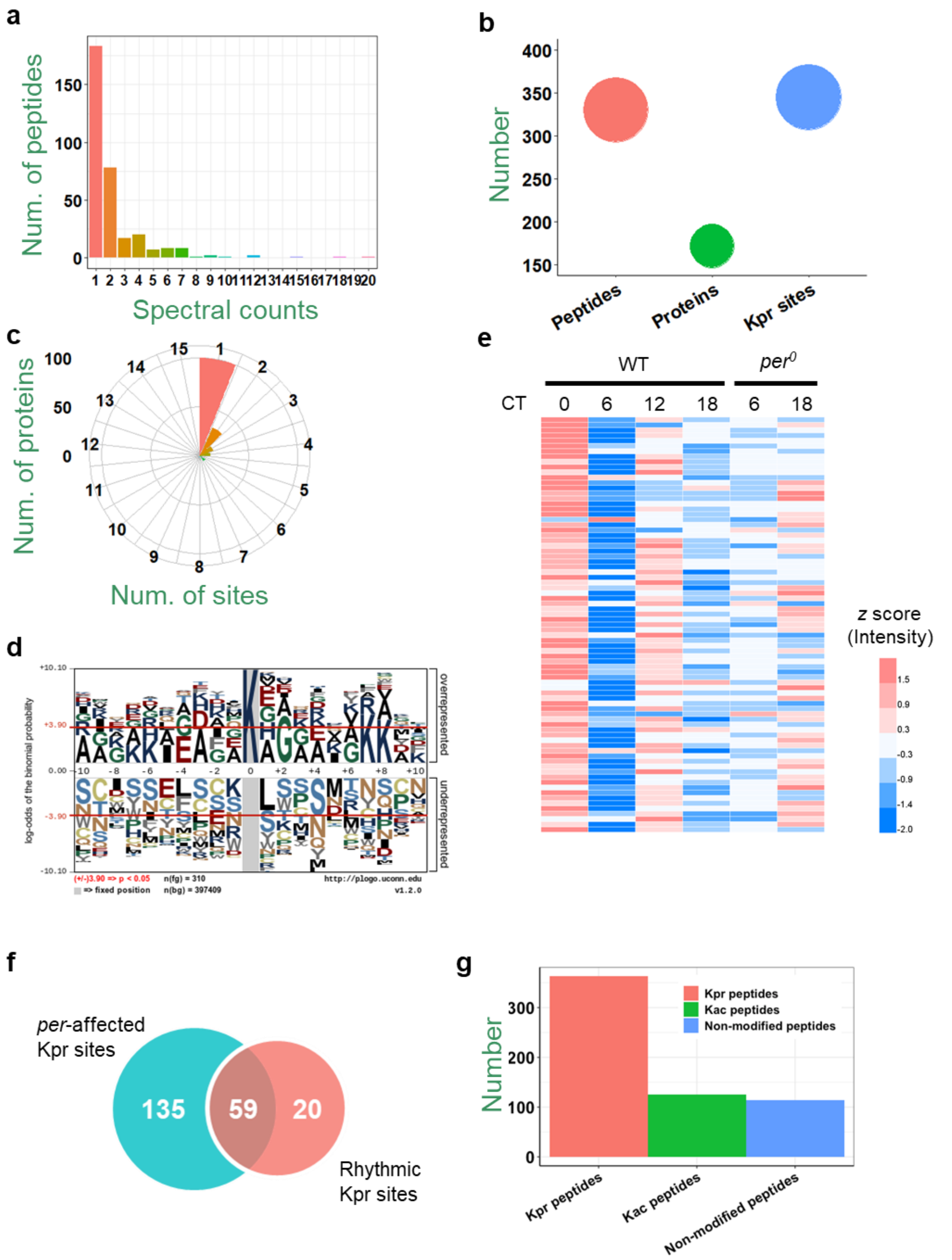

Supplementary Fig. 1: Propionylomic data analysis.

a The distribution of raw MS/MS spectral counts of propionylated peptides.

b The numbers of propionylated peptides, proteins and lysine sites identified in this study.

c The distribution of Kpr sites on propionylated proteins.

d Sequence analysis of propionylated peptides.

e The normalized propionylation intensities of 79 lysine sites with > 1.5 FC change throughout the day in WT flies.

f The 79 Kpr sites showing >1.5 FC (rhythmic Kpr sites) *vs*. sites at which propionylation level is affected by per^0 mutation (*per*-affected Kpr sites).

g Number of Kpr, Kac and non-modified peptides identified with both propionylation and acetylation selected as variable modification during database search.

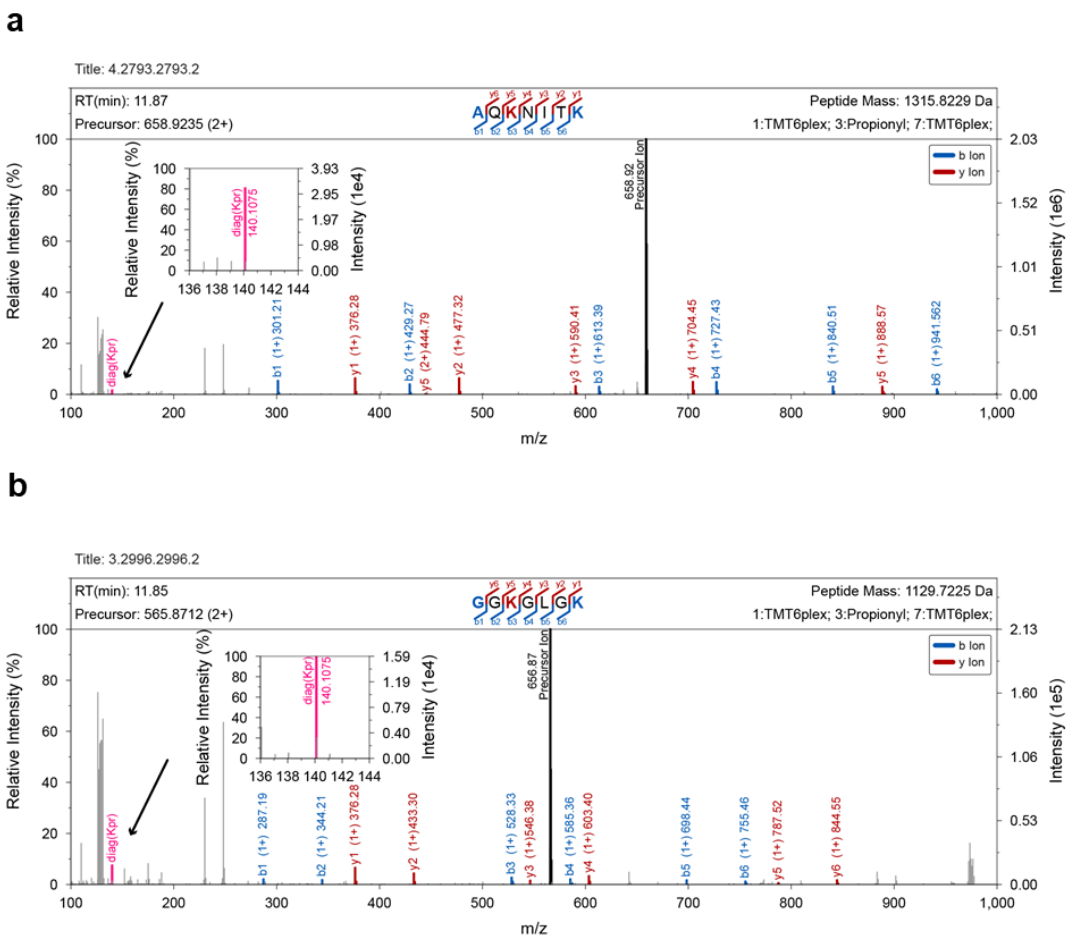

Supplementary Fig. 2: Examples of the diagnostic ion for Kpr in propionylated peptides.

a, **b** The MaxQuant MS/MS spectrums of H2B AQK(pr)NITK peptide (**a**) and H4 GGK(pr)GLGK peptide (**b**) identified in fly heads. The propionylated K corresponds to K17 in H2B (**a**) and K8 in H4 (**b**).

The values of *m*/*z* in MS/MS spectra are provided as a Source Data file.

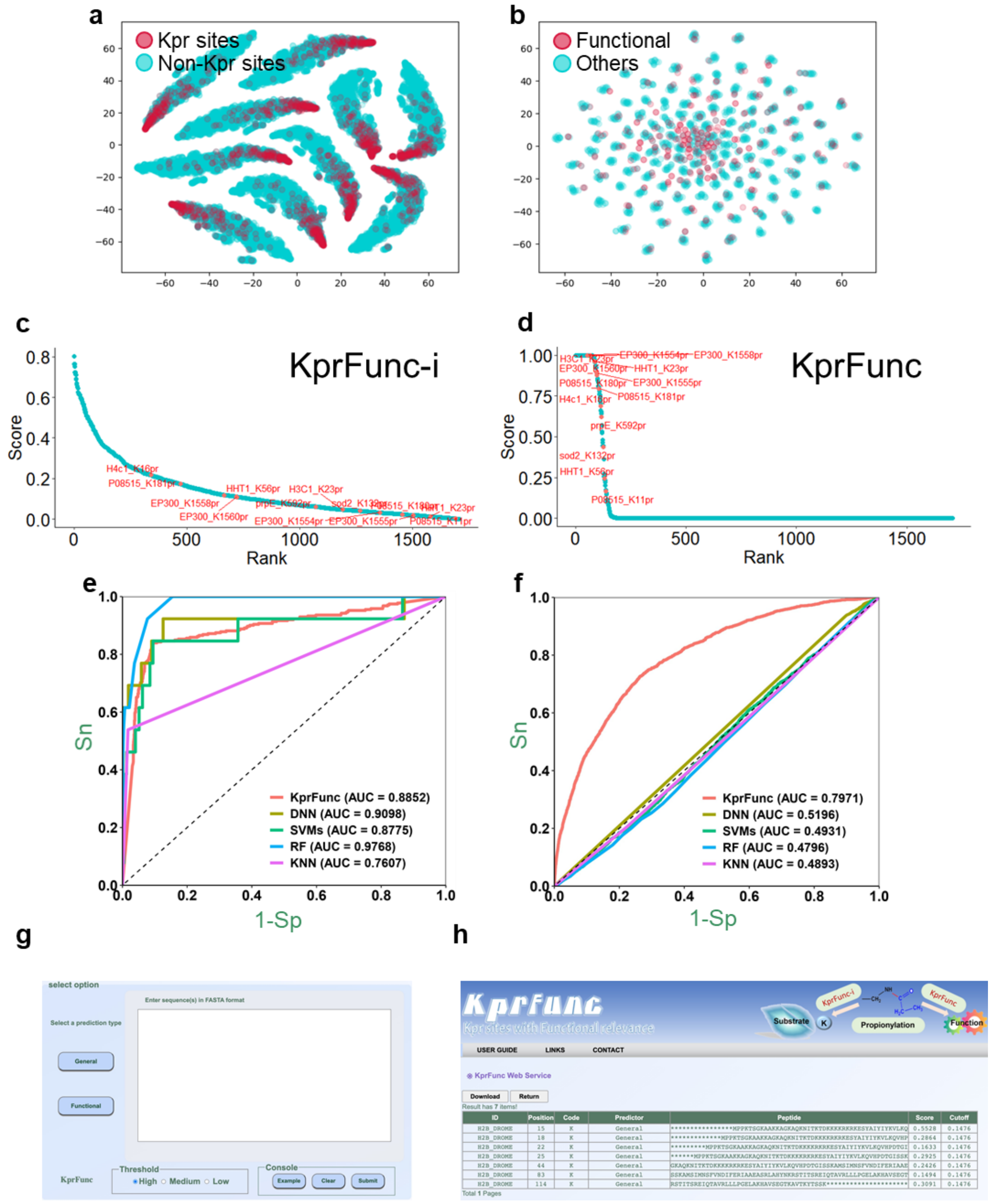

Supplementary Fig. 3: Performance analysis of KprFunc.

a The t-SNE analysis of Kpr sites and non-propionylated lysine residues separated by KprFunc-i.

b The t-SNE analysis of functional Kpr sites and other Kpr sites separated by KprFunc.

c, **d** Ranking of 1,707 Kpr sites scored by KprFunc-i (**c**) and KprFunc (**d**), with 13 known functional Kpr sites labeled.

e ROC curves and AUC values of 5-fold cross-validations of other methods for the prediction of functional Kpr sites.

f ROC curves and AUC values demonstrating the performance of KprFunc and other methods in distinguishing general Kpr sites from non-Kpr sites.

g Online service of KprFunc (http://kprfunc.biocuckoo.cn/).

h Prediction results of KprFunc, including protein accession number/ID, propionylation position, residue type, predictor used, flanking peptide, predicted score, and pre-defined cut-off value.

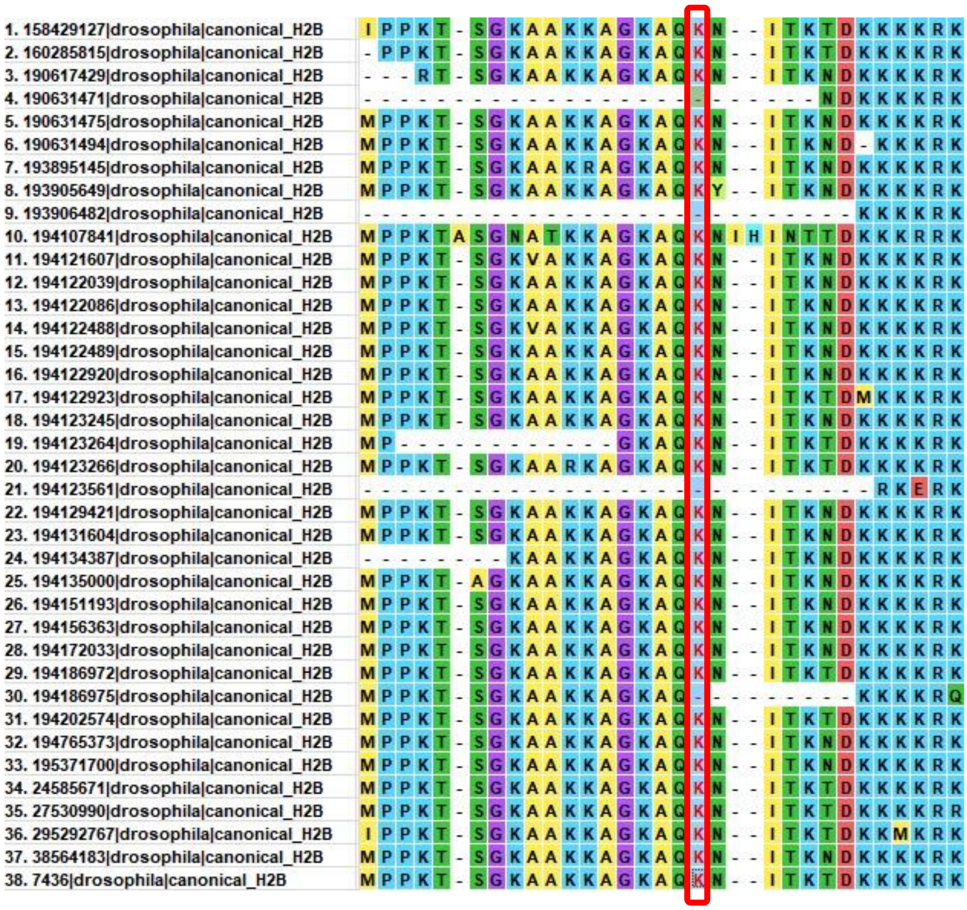

Supplementary Fig. 4: Sequence analysis of H2BK17.

Multiple sequence alignment of 38 canonical H2Bs in *Drosophila*, and K17 is marked in red.

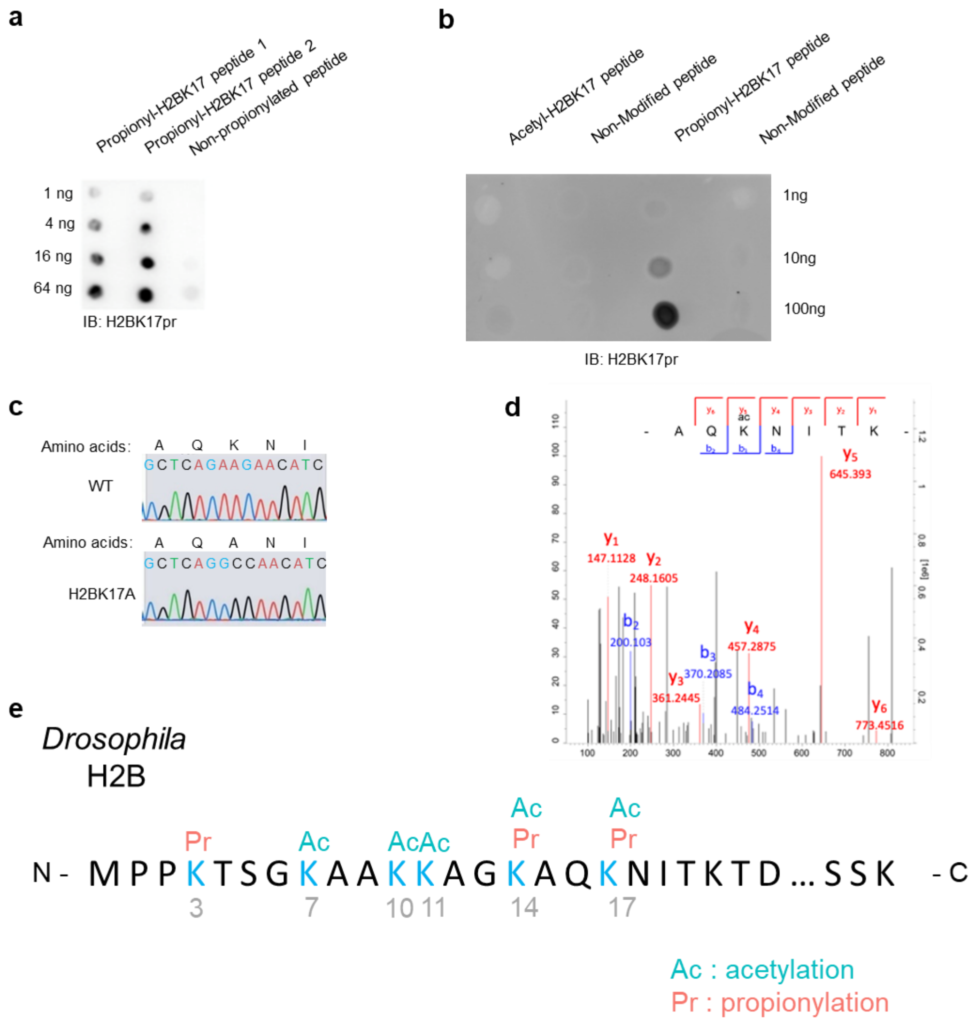

Supplementary Fig. 5: Generation and validation of H2BK17pr antibody.

a Specificity of H2BK17pr antibody is determined by dot blot assay. Indicated load of propionylated *vs.* unpropionylated peptides were blotted with H2BK17pr antibody. IB, immunoblot.

b Specificity of H2BK17pr antibody is determined by dot blot assay. Indicated load of propionylated *vs.* acetylated peptides and unpropionylated peptides were blotted with H2BK17pr antibody. IB, immunoblot.

c The sequencing traces of WT and H2BK17A flies confirming the H2BK17A mutation.

d The MaxQuant MS/MS spectra of AQK(ac)NITK peptide identified in fly heads. The acetylated K corresponds to H2BK17.

e Distribution of H2B lysine acetylation (Ac) and propionylation (Pr) identified in fly heads via immunoprecipitation with the H2BK17pr antibody followed by MS. Amino

acid sequences are shown with the acylated lysine residues in bold.

The blotting experiments were conducted with three independent repeats (**a** and **b**). Source data are provided as a Source Data file.

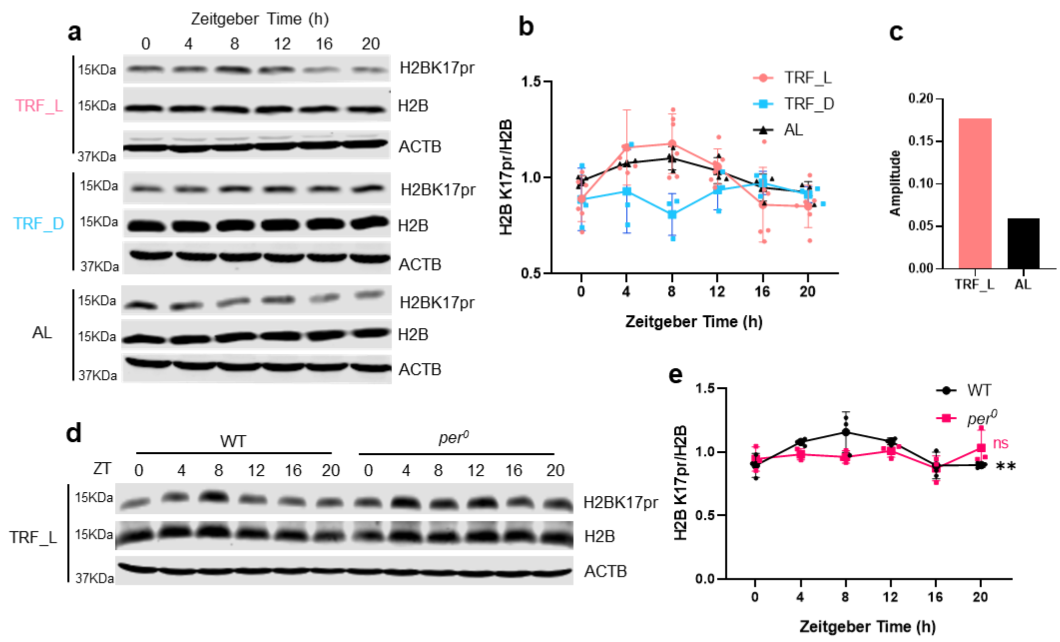

Supplementary Fig. 6: H2BK17pr oscillation is driven by daytime TRF in a circadiana clock dependent manner.

a Representative Western blots of proteins from whole-head extracts of flies under different feeding regime. Fly heads are collected at the indicated ZT during LD. TRF_L, feeding restricted to the light phase; TRF_D, feeding restricted to the dark phase; AL, *ad libitum* feeding.

b Plot shows quantification of normalized H2BK17pr level of blots in (**a**). The normalized intensity at AL ZT0 is set to 1. Statistical significance was calculated with JTK_CYCLE (TRF_L, n = 6 biologically independent experiments, ***p = 0.000017; TRF_D, n = 3 biologically independent experiments, p = 1; AL, n = 3 biologically independent experiments, *** p = 0.000167). ACTB is used as a loading control. ns, not significant.

c Plot shows amplitude of significant oscillation calculated by JTK_CYCLE in (**b**).

d Representative Western blots of proteins from whole-head extracts of WT and per^0 flies treated with feeding restricted to the light phase and collected at the indicated ZT during LD.

e Plot shows quantification of normalized H2BK17pr level of blots in (**d**). The average intensity of WT at ZT0 is set to 1 (n = 3 biologically independent experiments). Statistical significance was calculated with JTK_CYCLE: WT, ** p = 0.004117; per^0,

$p = 1$).

Data are presented as the mean ± SD. The blotting experiments were conducted with at least three independent repeats with similar results (**a** and **d**). Source data are provided as a Source Data file.

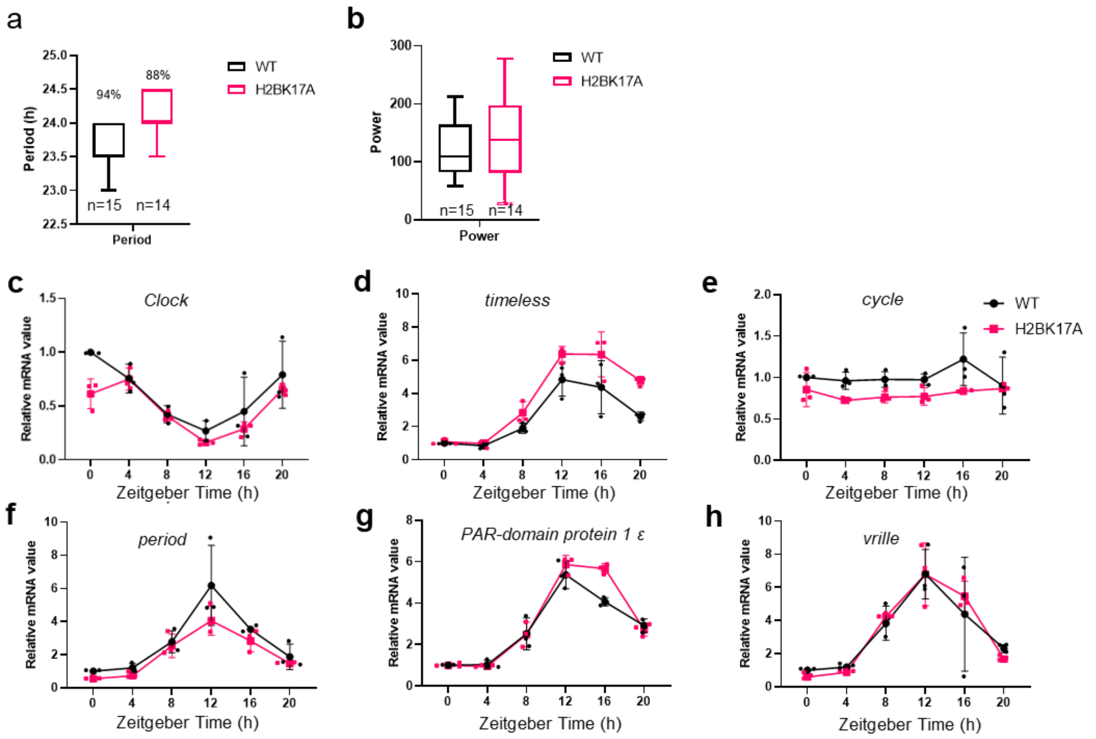

Supplementary Fig. 7: H2BK17A does not affect locomotor rhythm or the molecular clock.

a, **b** Box and whisker plots present the means (lines inside the boxes), the 5 and 95 percentile (bottom and top bounds of the boxes), and the extents (whiskers) of the period (**a**) and power (**b**) of DD locomotor rhythms. Number of flies is indicated on the plots (two-tailed Mann-Whitney *U* test for unpaired comparisons).

c-h Plots show the relative mRNA abundance of core circadian clock genes *Clock* (**c**), *timeless* (**d**), *cycle* (**e**), *per* (**f**), *PAR-domain protein 1 ε* (**g**) and *vrille* (**h**) determined by qRT-PCR from whole-head total RNA extracts of WT and H2BK17A flies collected at indicated ZT. For each plot, the average value of WT at ZT0 is set to 1 (n = 3 biologically independent experiments, two-tailed Mann-Whitney *U* test for unpaired comparisons).

Data are presented as the mean ± SD. Source data are provided as a Source Data file.

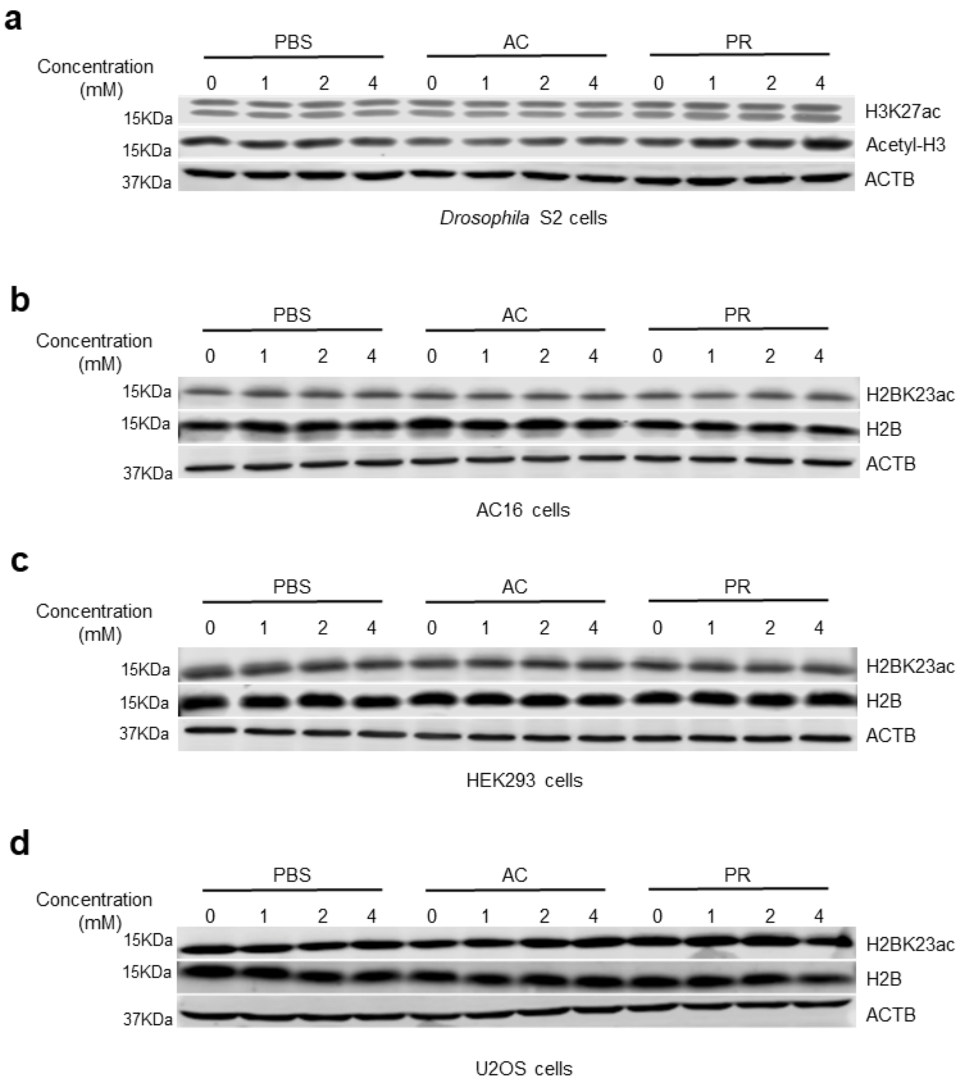

Supplementary Fig. 8: Propionate treatment does not exert substantial effect on acetylation level.

a-d Representative Western blots of proteins from *Drosophila* S2 cells (**a**), AC16 cells (**b**) HEK293 cells (**c**) and U2OS cells (**d**) treated with indicated concentration of sodium acetate (AC), sodium propionate (PR) or PBS for 24 h. ACTB is used as a loading control.

The blotting experiments were conducted with at least three independent repeats with similar results. Source data are provided as a Source Data file.

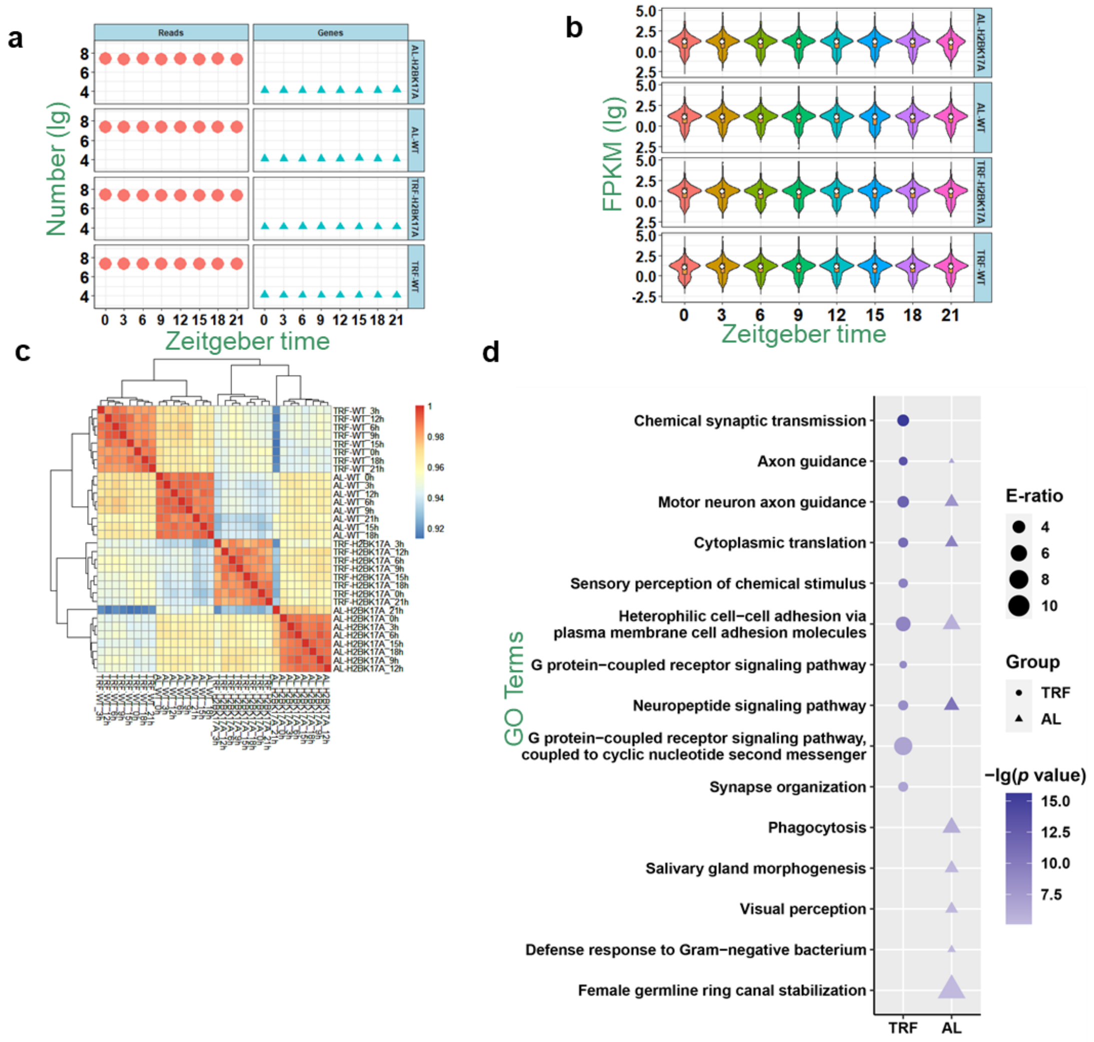

Supplementary Fig. 9: RNA-seq data analysis.

a Two-way hierarchical clustering of all RNA-seq samples.

b The number of raw reads and mapped genes in each RNA-seq sample.

c The FPKM values for mapped genes in each sample.

d GO-based enrichment analysis of DEGs in H2BK17A flies compared to WT under AL and TRF conditions (one-sided hypergeometric test).

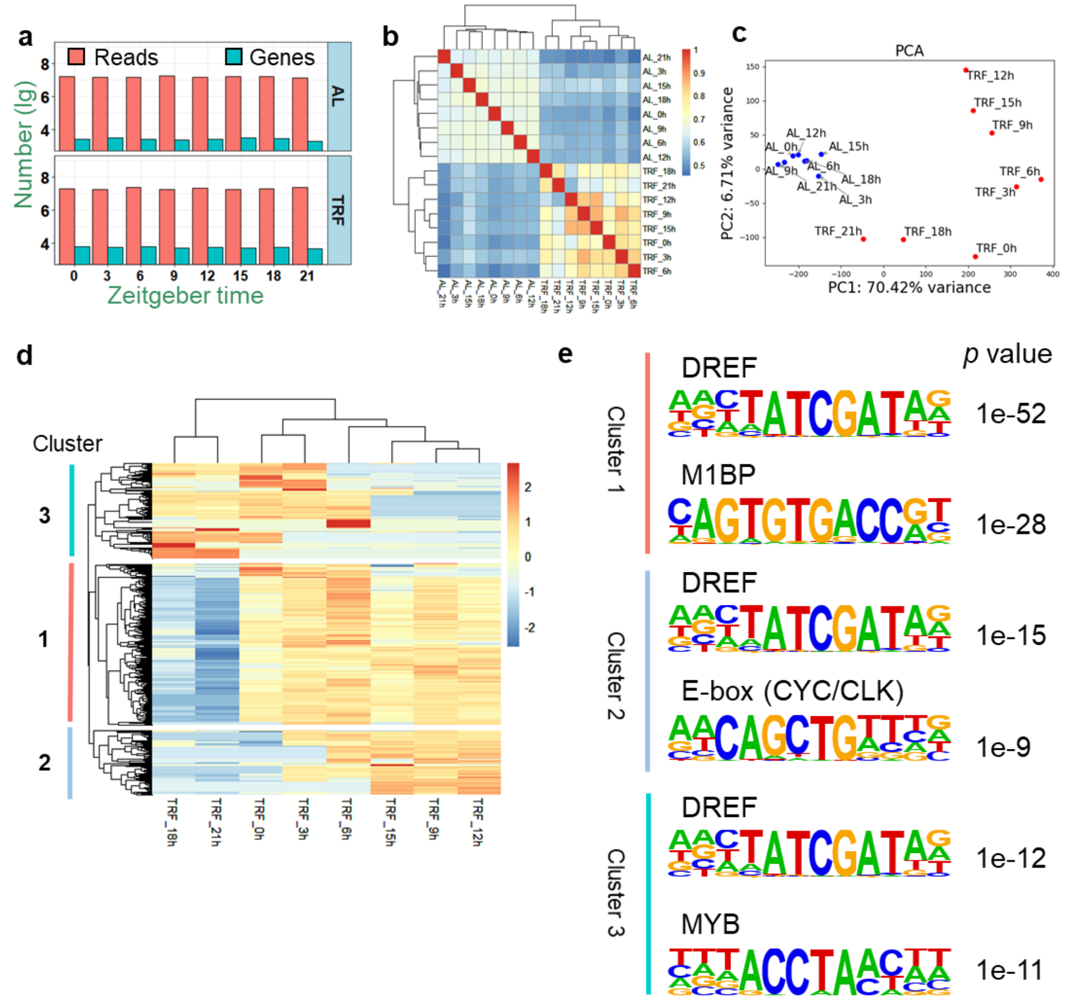

Supplementary Fig. 10: H2BK17pr ChIP-seq analysis.

a The number of raw reads and mapped genes in each ChIP-seq sample under AL- and TRF- treatments.

b Two-way hierarchical clustering of all ChIP-seq samples collected at the indicated ZT.

c Principle component analysis (PCA) of ChIP-seq samples collected at the indicated ZT. PC, principle component.

d Two-way hierarchical clustering of 1,386 genes rhythmically bound by H2BK17pr.

e *Drosophila* transcription factor motifs enriched in different clusters in (**d**).

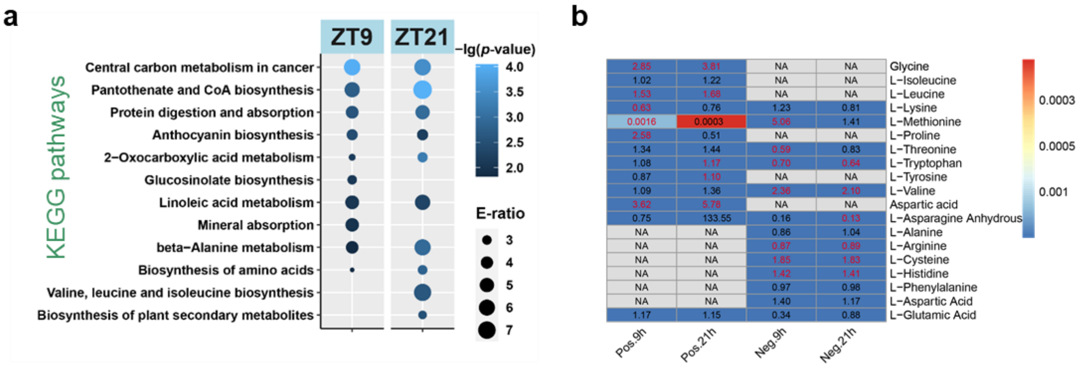

Supplementary Fig. 11: Metabolomics data analysis.

a KEGG-based enrichment analysis of metabolites with significant increase in TRF-WT samples compared to TRF-H2BK17A samples collected at ZT9 and ZT21. E-ratio, enrichment ratio (one-sided Hypergeometric test).

b Fold change of positive (POS) and negatively (NEG) charged amino acid metabolites identified in TRF-WT samples compared to TRF-H2BK17A samples collected at ZT9 and ZT21. Significant changes are labeled in red.

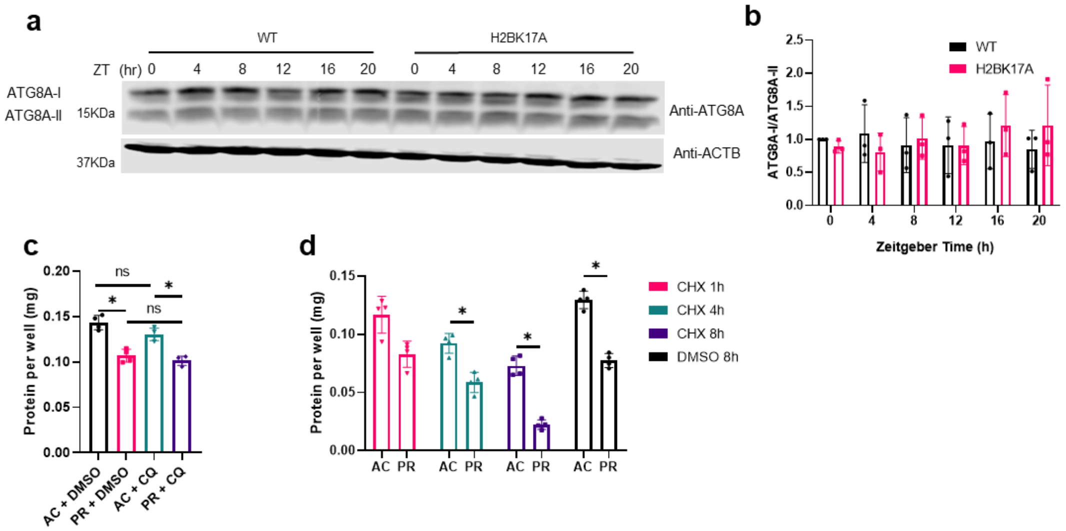

Supplementary Fig. 12: H2BK17pr does not appear to regulate global protein level via ALS or translation.

a Representative Western blots of proteins from whole-head extracts of WT and H2BK17A flies and collected at the indicated ZT during LD. ACTB is used as a loading control. The blotting experiments were conducted with at least three independent repeats with similar results.

b Histogram shows quantification of normalized ATG8A-I/ATG8A-II level of blots in **a**. The average intensity of WT at ZT0 is set to 1 (n = 3 biologically independent experiments; two-tailed Mann-Whitney U test for unpaired comparisons).

c Histogram shows total protein level of S2 cells treated with 5 mM sodium propionate (PR), 5 mM sodium acetate (AC), dimethyl sulfoxide (DMSO) and chloroquine (CQ) for 48h (n = 4 biologically independent experiments, two-tailed Mann-Whitney U test for unpaired comparisons, * p = 0.02857).

d Histogram shows total protein level of S2 cells treated with 5 mM sodium propionate (PR), 5 mM sodium acetate (AC), DMSO and cycloheximide (CHX) for indicated time durations (n = 4 biologically independent experiments, two-tailed Mann-Whitney U test for unpaired comparisons, * p = 0.02857).

Data are presented as the mean ± SD. Source data are provided as a Source Data file.

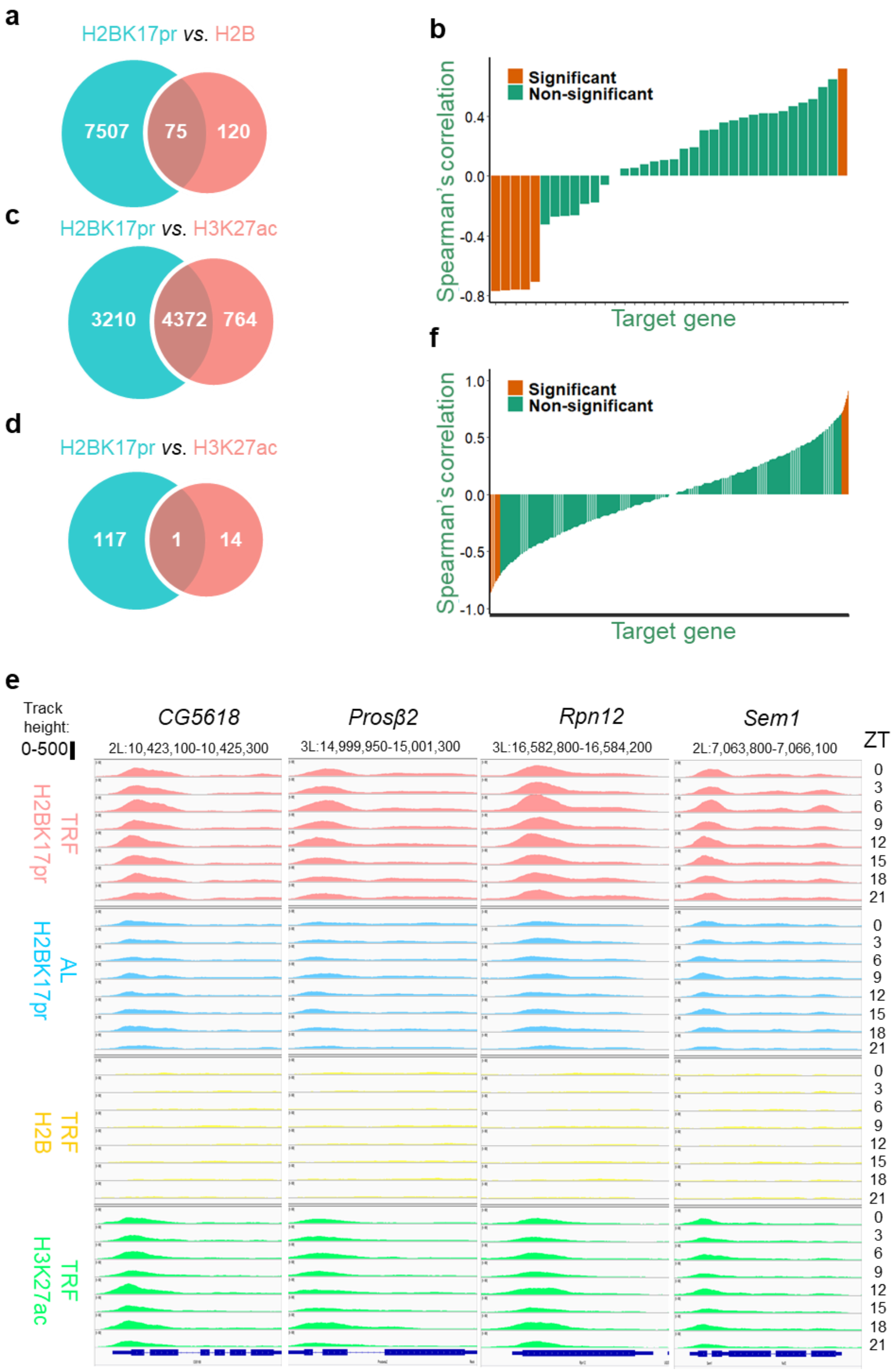

Supplementary Fig. 13: H2B and H3K27ac ChIP-seq analysis.

a Number of genes occupied by H2BK17pr *vs.* H2B under TRF.

b Spearman's correlation between 36 genes with both H2BK17pr and H2B binding peaks at 3 or more time points.

c Number of genes occupied by H2BK17pr *vs.* H3K27ac under TRF.

d Number of genes that are rhythmically expressed and rhythmically occupied by H2BK17pr *vs.* H3K27ac under TRF.

e Integrative Genomics Viewer (IGV) snapshots depicting H2BK17pr, H2B and H3K27ac binding peaks at indicated genes under TRF and AL condition.

f Spearman's correlation between 3,846 genes with both H2BK17pr and H3K27ac binding peaks at 3 or more time points.

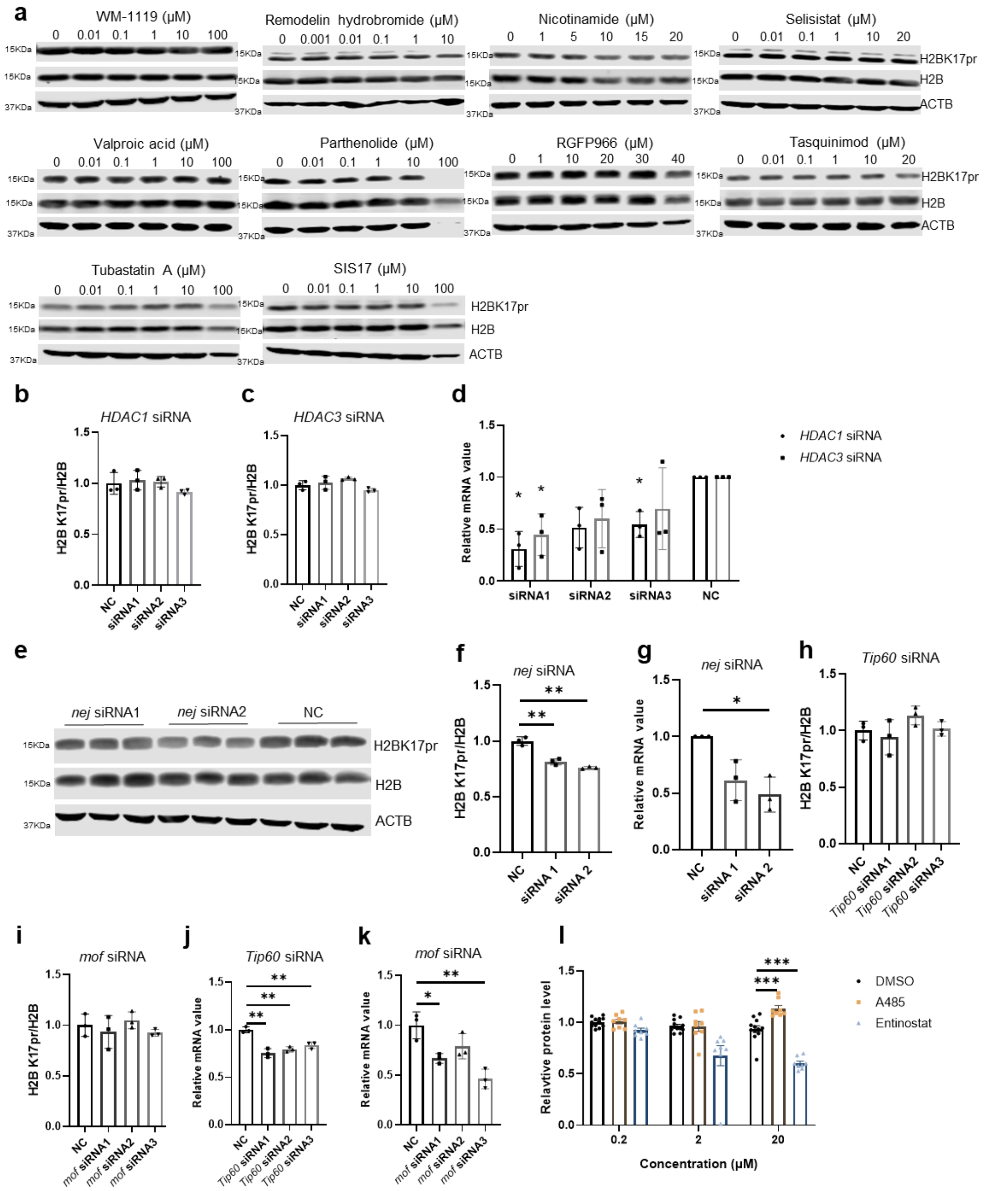

Supplementary Fig. 14: Screening for KATs and KDACs involved in regulating H2BK17pr.

a Western blots of protein extracts from S2 cells treated with indicated inhibitors under indicated concentrations for 24 h.

b, **c** Histograms show normalized H2BK17pr level in cells transfected with siRNA against *HDAC1* (**b**) and *HDAC3* (**c**) or negative control ($n = 3$ biologically independent experiments, two-tailed Student's t test).

d Histogram shows the mRNA level of *HDAC1/3* in cells transfected with siRNA

against *HDAC1/3* (*n* = 3, two-tailed Student's *t* test, from left to right, **p* = 0.01928, 0.02441 and 0.04085).

e Representative Western blots of protein extracts from S2 cells transfected with siRNA against *nej*.

f Histograms show normalized H2BK17pr level in cells transfected with siRNA against *nej* or negative control in (**e**). (*n* = 3 biologically independent experiments, two-tailed Student's *t* test, **p* = 0.028892).

g Histograms show the mRNA level of *nej* in cells transfected with siRNA against *nej* or negative control (*n* = 3 biologically independent experiments, two-tailed Student's *t* test, **p* = 0.02889).

h, **i** Histograms show normalized H2BK17pr level in cells transfected with siRNA against *Tip60* (**h**) and *mof* (**i**) or negative control (*n* = 3 biologically independent experiments, two-tailed Student's *t* test).

j Histogram shows the mRNA level of *Tip60* in cells transfected with siRNA against *Tip60* (*n* = 3 biologically independent experiments, two-tailed Student's *t* test, from left to right, ***p* = 0.003807, 0.001034 and 0.002872 from left to right).

k Histogram shows the mRNA level of *mof* in cells transfected with siRNA against *mof* (*n* = 3 biologically independent experiments, two-tailed Student's *t* test, **p* = 0.037882, ***p* = 0.006387).

l Histogram shows relative protein level of S2 cells treated with A485 and Entinostat for 24 h at indicated concentrations with DMSO as the control (two-tailed Student's *t* test. DMSO, *n* = 12 biologically independent experiments; A485, *n* = 8 biologically independent experiments, ****p* = 0.0004; Entinostat, *n* = 8 biologically independent experiments, ****p* = 4.96E-07). ACTB is used as a loading control. NC, negative control. The normalized intensity of negative control is set to 1. Data are presented as the mean ± SD.

The blotting experiments were conducted with at least three experiments with similar results (**a** and **e**). Source data are provided as a Source Data file.

Supplementary References

1. Powers ET, Morimoto RI, Dillin A, Kelly JW, Balch WE. Biological and chemical approaches to diseases of proteostasis deficiency. *Annu Rev Biochem* **78**, 959-991 (2009).
2. Hetz C. Adapting the proteostasis capacity to sustain brain healthspan. *Cell* **184**, 1545-1560 (2021).
3. Hipp MS, Kasturi P, Hartl FU. The proteostasis network and its decline in ageing. *Nat Rev Mol Cell Biol* **20**, 421-435 (2019).
4. Sebastian RM, Shoulders MD. Chemical Biology Framework to Illuminate Proteostasis. *Annu Rev Biochem* **89**, 529-555 (2020).
5. Inagi R, Ishimoto Y, Nangaku M. Proteostasis in endoplasmic reticulum--new mechanisms in kidney disease. *Nat Rev Nephrol* **10**, 369-378 (2014).
6. Klaips CL, Jayaraj GG, Hartl FU. Pathways of cellular proteostasis in aging and disease. *J Cell Biol* **217**, 51-63 (2018).
7. Zhou D, Palam LR, Jiang L, Narasimhan J, Staschke KA, Wek RC. Phosphorylation of eIF2 directs ATF5 translational control in response to diverse stress conditions. *J Biol Chem* **283**, 7064-7073 (2008).
8. Nitika, Porter CM, Truman AW, Truttmann MC. Post-translational modifications of Hsp70 family proteins: Expanding the chaperone code. *J Biol Chem* **295**, 10689-10708 (2020).
9. Trepel J, Mollapour M, Giaccone G, Neckers L. Targeting the dynamic HSP90 complex in cancer. *Nat Rev Cancer* **10**, 537-549 (2010).
10. Muller P*, et al.* C-terminal phosphorylation of Hsp70 and Hsp90 regulates alternate binding to co-chaperones CHIP and HOP to determine cellular protein folding/degradation balances. *Oncogene* **32**, 3101-3110 (2013).
11. Glotzer M, Murray AW, Kirschner MW. Cyclin is degraded by the ubiquitin pathway. *Nature* **349**, 132-138 (1991).
12. King RW, Deshaies RJ, Peters JM, Kirschner MW. How proteolysis drives the cell cycle. *Science* **274**, 1652-1659 (1996).
13. Lienhard GE. Non-functional phosphorylations? *Trends Biochem Sci* **33**, 351-352 (2008).
14. Landry CR, Levy ED, Michnick SW. Weak functional constraints on phosphoproteomes. *Trends Genet* **25**, 193-197 (2009).
15. Beltrao P*, et al.* Systematic functional prioritization of protein posttranslational modifications. *Cell* **150**, 413-425 (2012).
16. Ochoa D*, et al.* The functional landscape of the human phosphoproteome. *Nat Biotechnol* **38**, 365-373 (2020).
17. Li WJ*, et al.* Insulin signaling regulates longevity through protein phosphorylation in Caenorhabditis elegans. *Nat Commun* **12**, 4568 (2021).
18. Finn C, Abbeel P, Levine S. Model-agnostic meta-learning for fast adaptation of deep networks. In: *International conference on machine learning*). PMLR (2017).
19. Ju Z, He JJ. Prediction of lysine propionylation sites using biased SVM and incorporating four different sequence features into Chou's PseAAC. *J Mol*

Graph Model **76**, 356-363 (2017).

20. Xu H, Zhou J, Lin S, Deng W, Zhang Y, Xue Y. PLMD: An updated data resource of protein lysine modifications. *J Genet Genomics* **44**, 243-250 (2017).

21. Zhang W*, et al.* CPLM 4.0: an updated database with rich annotations for protein lysine modifications. *Nucleic Acids Res* **50**, D451-D459 (2022).

22. Wang LN, Shi SP, Wen PP, Zhou ZY, Qiu JD. Computing Prediction and Functional Analysis of Prokaryotic Propionylation. *J Chem Inf Model* **57**, 2896-2904 (2017).

23. Li A, Deng Y, Tan Y, Chen M. A Transfer Learning-Based Approach for Lysine Propionylation Prediction. *Front Physiol* **12**, 658633 (2021).
