# Supplementary material for: Small-sample learning reveals propionylation in determining global protein homeostasis
Source: Nat Commun. 2023 May 17;14:2813. doi: 10.1038/s41467-023-38414-8 (PMC10192394; doi:10.1038/s41467-023-38414-8)
Supplement: Supplementary file 3 — Description of Additional Supplementary Files [file 41467_2023_38414_MOESM3_ESM.pdf]

Description of Additional Supplementary Files

File Name: Supplementary Data 1
Description: Propionylomic data.

File Name: Supplementary Data 2
Description: All data sets related to KprFunc.

File Name: Supplementary Data 3
Description: RNA-seq related data.

File Name: Supplementary Data 4
Description: ChIP-seq related data.

File Name: Supplementary Data 5
Description: Metabolomic data.

File Name: Supplementary Data 6
Description: KATs and KDACs in Drosophila and their human orthologs.

File Name: Supplementary Data 7
Description: Detailed information regarding the H2BK17pr centered signal web.

File Name: Supplementary Data 8
Description: The key resources table.
