# Supplementary material for: Small-sample learning reveals propionylation in determining global protein homeostasis
Source: Nat Commun. 2023 May 17;14:2813. doi: 10.1038/s41467-023-38414-8 (PMC10192394; doi:10.1038/s41467-023-38414-8)
Supplement: Supplementary file 12 — Reporting Summary [file 41467_2023_38414_MOESM12_ESM.pdf]

nature portfolio

Corresponding author(s): Yu Xue, Luoying Zhang

Last updated by author(s): Mar 26, 2023

Reporting Summary

Nature Portfolio wishes to improve the reproducibility of the work that we publish. This form provides structure for consistency and transparency in reporting. For further information on Nature Portfolio policies, see our Editorial Policies and the Editorial Policy Checklist.

Statistics

For all statistical analyses, confirm that the following items are present in the figure legend, table legend, main text, or Methods section.

n/a	Confirmed	
☐	☒	The exact sample size (*n*) for each experimental group/condition, given as a discrete number and unit of measurement
☐	☒	A statement on whether measurements were taken from distinct samples or whether the same sample was measured repeatedly
☐	☒	The statistical test(s) used AND whether they are one- or two-sided<br>*Only common tests should be described solely by name; describe more complex techniques in the Methods section.*
☒	☐	A description of all covariates tested
☒	☐	A description of any assumptions or corrections, such as tests of normality and adjustment for multiple comparisons
☐	☒	A full description of the statistical parameters including central tendency (e.g. means) or other basic estimates (e.g. regression coefficient) AND variation (e.g. standard deviation) or associated estimates of uncertainty (e.g. confidence intervals)
☐	☒	For null hypothesis testing, the test statistic (e.g. F, t, r) with confidence intervals, effect sizes, degrees of freedom and P value noted<br>*Give P values as exact values whenever suitable.*
☒	☐	For Bayesian analysis, information on the choice of priors and Markov chain Monte Carlo settings
☒	☐	For hierarchical and complex designs, identification of the appropriate level for tests and full reporting of outcomes
☐	☒	Estimates of effect sizes (e.g. Cohen's *d*, Pearson's *r*), indicating how they were calculated

Our web collection on statistics for biologists contains articles on many of the points above.

Software and code

Policy information about availability of computer code

Data collection	Q-Exactive-plus mass spectrometer (Thermo Fisher Scientific) was used to collect all proteomics data.<br>Illumina NovaSeq 6000 platform (Novogene, Tianjing, China) was used to collect RNA-seq and ChIPseq data; Reads were aligned to the Drosophila genome and IGV (v2.5.0, NCI, US) was used to visualization.<br>Raw reads of RNA-seq were used to generate the BAM files by software packages of Bowtie2 (version 2.2.4) and TopHat (version 2.1.1), Cufflinks (version 2.2.1) was employed to assemble the reads and calculate the expression levels of individual mRNAs based on FPKM values. MaxQuant (v.1.5.3.30) was used for standard database search of MS/MS raw data against Drosophila proteome database downloaded from UniProt (Version 201706). For analysis of the ChIP-seq data, Bowtie2 (version 2.2.4) was adopted for reads mapping to the reference genome of D. melanogaster downloaded from Ensembl. Samtools (version 1.3.1) was used to sort the BAM files and generate the SAM files. The peak calling was performed by MACS2 (version 2.1.4), and peak annotation was conducted with ChIPseeker. Identified peaks from different time points were merged by the program mergePeaks in HOMER (version 4.11).The raw files of metabolomic profiling was taken as the input of ProteoWizard software package (version 3.0 https://proteowizard.sourceforge.io/) for peak extraction. Alignment and retention time correction was conducted using the R package XCMS program.<br>Images of WB was acquired with a Image Studio (v4.0,LI-COR Biosciences, US). SkanIt for Multiskan GO software (v3.1, Thermo Fisher Scientific) was used to measure light absorbance.<br>Stepone Software (v2.3, Applied Biosystems) was used to collect and analysis qRT-PCR data.
Data analysis	For model training of KprFunc, logistic regression was performed with scikit-learn (version 0.21.0), and DNN framework was construded using Keras (version 2.4.3). Circadian oscillations at different levels were identified by MetaCycle. PyMol 2.5.0 (https://pymol.org/2/), was used for visualization of fly H2B and calculation of distances between H2BK17pr and other residues. The multi-alignment of all sequences was performed by MEGA 7.0.26. PyMol 2.5.0 (https://pymol.org/2/) was used for visualization of fly H2B and calculation of distances between H2BK17pr and other residues., The program findMotifsGenome in HOMER (version 4.11) was used for the motif enrichment analysis. The transcriptional regulations between TFs and genes regulated by H2BK17pr were predicted by the fimo program in MEME Suite 4.10.0.<br>The source code of KprFunc has been uploaded to GitHub (https://github.com/CuckooWang/KprFunc) with the DOI identifier (https://

doi.org/ 10.5281/zenodo.4798325)

For manuscripts utilizing custom algorithms or software that are central to the research but not yet described in published literature, software must be made available to editors and reviewers. We strongly encourage code deposition in a community repository (e.g. GitHub). See the Nature Portfolio guidelines for submitting code & software for further information.

Data

Policy information about availability of data

All manuscripts must include a data availability statement. This statement should provide the following information, where applicable:

- Accession codes, unique identifiers, or web links for publicly available datasets
- A description of any restrictions on data availability
- For clinical datasets or third party data, please ensure that the statement adheres to our policy

The raw ChIP-seq and RNA-seq data have been deposited to the National Genomics Data Center (NGDC, https://ngdc.cncb.ac.cn/) with the data set identifier PRJCA007845. The raw MS/MS data of propionylomic and metabolomic profiles have been deposited to the integrated proteome resources (iProX, https://www.iprox.org/). All annotated MS/MS spectra of propionylomic profiling were also provided. The accession number of the MS/MS data reported in this paper is IPX0003936000, and associated ProteomeXchange accession code is PXD040987. Source data are provided with this paper. Eukaryotic H2B protein sequences were downloaded from database HistoneDB 2.0 (https://www.ncbi.nlm.nih.gov/research/HistoneDB2.0/index.fcgi/browse/). D. melanogaster proteome database was obtained from UniProt (https://www.uniprot.org/ , Version 201706). Details of propionyl modification was acquired from database Unimod (http://www.unimod.org/). Partial Kpr sites used for model training came from previously developed database CPLM 4.0 (http://cplm.biocuckoo.cn/). The predicted 3D structure of fly H2B protein was downloaded from AlphaFold Protein Structure Database (https://www.alphafold.ebi.ac.uk/) as a PDB file. UPS enzymes/regulators and ALS regulators incorporated in this study were extracted from database iUUCD (http://iuucd.biocuckoo.org/) and THANANTOS (http://thanatos.biocuckoo.org/), respectively. TFs of H. sapiens and D. melanogaster and corresponding classifications were downloaded from database AnimalTFDB 3.0 (http://bioinfo.life.hust.edu.cn/AnimalTFDB/#!/).

Field-specific reporting

Please select the one below that is the best fit for your research. If you are not sure, read the appropriate sections before making your selection.

☒ Life sciences ☐ Behavioural & social sciences ☐ Ecological, evolutionary & environmental sciences

For a reference copy of the document with all sections, see nature.com/documents/nr-reporting-summary-flat.pdf

Life sciences study design

All studies must disclose on these points even when the disclosure is negative.

Sample size	Although there is no formal power calculation to define sample size, all cell and drosophila experiments were conducted independently with n =3+, and the sample size was chosen to be consistent with the previous literature by utilizing similar assays. Specific replicate numbers are indicated in the text. Exceptions included RNAseq, ChIPseq and Mass spectrometry where an extraordinarily large sample volume was required.
Data exclusions	No data was excluded.
Replication	At least 3 times biological replicates were performed as described in the main text.
Randomization	Flies were allocated based on the corresponding genotype. No randomization was performed for RNAseq, ChIPseq, mass spectrometry and in vitro experiments because whole samples were used. For the rest of in vivo experiments, flies were randomly allocated . For immunostaining, all genotypes were prepared in one session, stained in one tube and strictly analyzed in an unbiased manner.
Blinding	Investigators were not blinded to Drosophila genotypes during experiments because they are quantitative measurements that did not require subjective interpretation or judgement..

Reporting for specific materials, systems and methods

We require information from authors about some types of materials, experimental systems and methods used in many studies. Here, indicate whether each material, system or method listed is relevant to your study. If you are not sure if a list item applies to your research, read the appropriate section before selecting a response.

Materials & experimental systems

n/a	Involved in the study
☐	☒ Antibodies
☐	☒ Eukaryotic cell lines
☒	☐ Palaeontology and archaeology
☐	☒ Animals and other organisms
☒	☐ Human research participants
☒	☐ Clinical data
☒	☐ Dual use research of concern

Methods

n/a	Involved in the study
☐	☒ ChIP-seq
☒	☐ Flow cytometry
☒	☐ MRI-based neuroimaging

Antibodies

Antibodies used

The following primary antibodies were used: pan anti-propionylation (PTM-201, PTM BIO, CN), anti-H2BK17pr, anti-H2B (ab52484, Abcam, US), anti-H2BK23ac (PTM-174, PTM BIO, CN), anti-H3K27ac (ab4729, Abcam, US),anti-ACTB (AC026, ABclonal, CN), Anti-ubiquitination (sc-8017, SCBT, US), Anti-ATG8A (ab109364, Abcam, US), mouse normal IgG (sc-2025, SCBT, US), rabbit normal IgG (2729, CST, US). Secondary antibodies for Western blot were: IRDye 800CW donkey anti-rabbit (926-32213, LI-COR Biosciences, US), IRDye 680 donkey anti-mouse IgG antibody (926-68072, LI-COR Biosciences, US).

Validation

pan anti-propionylation (PTM-201, PTM BIO, CN): The antibody is validated at 1:2000 dilution for WB of unmodified BSA peptides vs. propionylated peptides and published for WB in multiple studies (e.g. PMID: 32010779).
anti-H2BK17pr: We generated this antibody that specifically detects this particular modification but not acetylated or non-modified peptide (Supplementary Fig. 5a and 5b). We also be able to detect H2BK17pr signal in WT fly heads, while a fly line carrying H2BK17A lysine mutation which eliminates propionylation at this site does not exhibit H2BK17pr signal (Fig. 3e and Supplementary Fig. 5c). Moreover, propionate treatment could increase H2BK17pr in a dose-dependent manner in fly S2 cells (Fig. 3f).
anti-H2B (ab52484, Abcam, US): The antibody is validated at 1:1000 dilution for immunocytochemistry and published in multiple studies (e.g. PMID: 26988139).
anti-H2BK23ac (PTM-174, PTM BIO, CN): he antibody is validated at 1:1000 dilution for WB of unmodified BSA peptides vs. acetylated peptides.
anti-H3K27ac (ab4729, Abcam, US): The antibody is validated at for WB/ChIPseq and published for WB/ChIPseq in multiple studies (e.g. PMID: 26280901).
anti-ACTB (AC026, ABclonal, CN): The antibody is validated at 1:100000 dilution for WB of and published for WB in multiple studies (e.g. PMID: 35585240).
Anti-ubiquitination (sc-8017, SCBT, US): The antibody is validated at 1:1000 dilution for WB/IP and published in multiple studies (e.g. PMID: 25551675).
Anti-ATG8A (ab109364, Abcam, US): The antibody is validated at 1:100 dilution for immunocytochemistry and published in multiple studies (e.g. PMID: 26761346).
IRDye 800CW donkey anti-rabbit (926-32213, LI-COR Biosciences, US): The antibody was isolated by affinity chromatography using antigens coupled to agarose beads. Based on ELISA, this antibody reacts with the heavy and light chains of rabbit IgG and with the light chains common to most rabbit immunoglobulins. This antibody has been tested by ELISA and/or solid-phase adsorbed to ensure minimal cross-reaction with bovine, chicken, goat, guinea pig, hamster, horse, human, mouse, rat, and sheep serum proteins, but the antibody may cross-react with immunoglobulins from other species. The conjugate has been specifically tested and qualified for Western blot and In-Cell Western Assay applications.
IRDye 680 donkey anti-mouse IgG antibody (926-68072, LI-COR Biosciences, US): The antibody was isolated from antisera by immunoaffinity chromatography using antigens coupled to agarose beads. Based on immunoelectrophoresis, the antibody reacts with the heavy chains on mouse IgG and with the light chains common to most mouse immuno-globulins. No reactivity was detected against non-immunoglobulin serum proteins. This antibody has been tested by ELISA and/or solid-phase adsorbed to ensure minimal cross-reaction with bovine, chicken, goat, guinea pig, Syrian hamster, horse, human, rabbit, and sheep serum proteins, but the antibody may cross-react with immunoglobulins from other species. The conjugate has been specifically tested and qualified for Western blot and In-Cell Western Assay applications.

Eukaryotic cell lines

Policy information about cell lines

Cell line source(s)

S2 cells were generous gifted from Dr. Xi Zhou (Wuhan University, China) and obtained from ATCC (CRL-1963). Mammalian U2OS (HTB-96), NIH-3T3 (CRL-1658) and HEK293 (CRL-1573) cells were obtained from ATCC.

Authentication

None of cell lines used were authenticated.

Mycoplasma contamination

Cell lines were not tested for mycoplasma contamination.

Commonly misidentified lines
(See ICLAC register)

No commonly misidentified cell lines used in the study.

Animals and other organisms

Policy information about studies involving animals; ARRIVE guidelines recommended for reporting animal research

Laboratory animals	For all experiments, male Drosophila melanogaster (fruit fly) within 1 week were used. Strains: w1118, H2BK17A, per0 and y w.
Wild animals	The study did not involve wild animals.
Field-collected samples	The study did not involve samples collected from the field.
Ethics oversight	The study was conducted using Drosophila melanogaster (fruit fly) and cell lines, therefore there is no ethical concern.

Note that full information on the approval of the study protocol must also be provided in the manuscript.

ChIP-seq

Data deposition

☒ Confirm that both raw and final processed data have been deposited in a public database such as GEO.

☒ Confirm that you have deposited or provided access to graph files (e.g. BED files) for the called peaks.

Data access links<br>*May remain private before publication.*	https://ngdc.cncb.ac.cn/gsa/s/048932G4
Files in database submission	PRJCA007845
Genome browser session<br>(e.g. UCSC)	N/A

Methodology

Replicates	No replicates were performed for the ChIPseq (n=1)
Sequencing depth	Sample Time Total reads<br>TRF 0h 19564519<br>TRF 3h 17170298<br>TRF 6h 23226415<br>TRF 9h 17422922<br>TRF 12h 20986346<br>TRF 15h 17757907<br>TRF 18h 20466015<br>TRF 21h 23121680<br>AL 0h 16077158<br>AL 3h 13894606<br>AL 6h 14781331<br>AL 9h 18030422<br>AL 12h 14281652<br>AL 15h 15574055<br>AL 18h 16711823<br>AL 21h 13220015<br>H2B_TRF ZT0 14612892<br>H2B_TRF ZT3 10332897<br>H2B_TRF ZT6 11865793<br>H2B_TRF ZT9 16783200<br>H2B_TRF ZT12 10799840<br>H2B_TRF ZT15 15163767<br>H2B_TRF ZT18 12820365<br>H2B_TRF ZT21 12652316<br>H3K27ac_TRF ZT0 12292670<br>H3K27ac_TRF ZT3 10636345<br>H3K27ac_TRF ZT6 12238397<br>H3K27ac_TRF ZT9 9963263<br>H3K27ac_TRF ZT12 14606740<br>H3K27ac_TRF ZT15 10376464<br>H3K27ac_TRF ZT18 15232177<br>H3K27ac_TRF ZT21 11047982
Antibodies	Anti-H2BK17pr, anti-H2B and anti-H3K27ac antibodies were used in ChIPseq.
Peak calling parameters	The peak calling was performed by MACS2 (version 2.1.4), and parameters were chosen as -g dm -f BAMPE -B -q 0.00001.

Data quality	q value < 0.00001
Software	The peak calling was performed by MACS2 (version 2.1.4), and parameters were chosen as -g dm -f BAMPE -B -q 0.00001.
